# Ultrasensitive colorimetric detection of creatinine *via* its dual binding affinity for silver nanoparticles and silver ions†

**DOI:** 10.1039/d3ra08736k

**Published:** 2024-03-18

**Authors:** Jingle Huang, Maria Sokolikova, Antonio Ruiz-Gonzalez, Yingqi Kong, Yuxuan Wang, Yingjia Liu, Lizhou Xu, Mingqing Wang, Cecilia Mattevi, Andrew Davenport, Tung-Chun Lee, Bing Li

**Affiliations:** a Institute for Materials Discovery, University College London London WC1H 0AJ UK bing.li@ucl.ac.uk tungchun.lee@ucl.ac.uk; b Department of Materials, Imperial College London London SW7 2AZ UK; c Zhejiang University-Hangzhou Global Scientific and Technological Innovation Centre Hangzhou 311200 China; d College of Biosystems Engineering and Food Science, Zhejiang University Hangzhou 310058 China; e Department of Renal Medicine, Royal Free Hospital, University College London NW3 2PF UK

## Abstract

Creatinine is an important biomarker for the diagnosis of chronic kidney disease (CKD). Recently, it has been reported that the concentration of salivary creatinine correlates well with the concentration of serum creatinine, which makes the former useful for the development of non-invasive and point-of-care (POC) detection for CKD diagnosis. However, there exists a technical challenge in the rapid detection of salivary creatinine at low concentrations of 3–18 μM when using the current kidney function test strips as well as the traditional methods employed in hospitals. Herein, we demonstrate a simple, sensitive colorimetric assay for the detection of creatinine with a limit-of-detection (LOD) down to the nanomolar level. Our approach utilises the dual binding affinity of creatinine for citrate-capped silver nanoparticles (Ag NPs) and Ag(i) ions, which can trigger the aggregation of Ag NPs and thus lead to the colour change of a sample. The quantitative detection of creatinine was achieved using UV-Vis spectroscopy with a LOD of 6.9 nM in artificial saliva and a linear dynamic range of 0.01–0.06 μM. This method holds promise to be further developed into a POC platform for the CKD diagnosis.

## Abstract 2

Creatinine detection *via* dual binding affinity for silver nanoparticles and silver ions.

## Graphical abstract

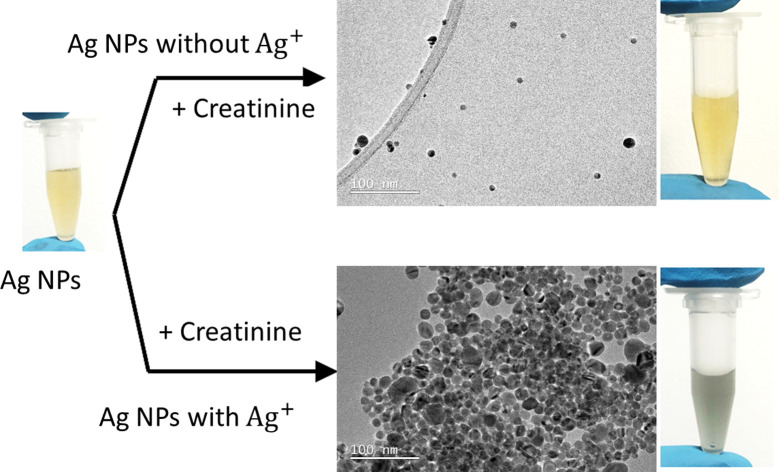

## 1. Introduction

About 10% of the global population is currently affected by CKD, which shows no symptom at its early stage, but could lead to kidney function failure, anaemia, metabolic acidosis, and increased risk of mortality at advanced stages.^1^ Hence, it is important to diagnose CKD at its early stage for the development of preventive strategies and effective treatments. Creatinine is a discriminative product that is filtered by kidneys from blood and excreted through urine. The blood creatinine level has been widely used as a biomarker for the diagnosis of renal diseases.^2,3^ However, blood sampling processes cause pain and discomfort, and the high precision analysis of creatinine relies on complicated equipment and skilled personnel, which make it challenging in resource-limited settings.^4^

Saliva is easy to acquire and store. Its collection process has a higher psychological acceptance compared with the collection of other body fluids.^5^ Thus, saliva is a promising alternative for the development of POC diagnostic methods.^6–8^ Salivary creatinine has been increasingly studied, which shows a diagnostic accuracy of 91%, compared with the diagnosis of CKD through blood creatinine.^9,10^ Similar to blood analysis, however, those techniques for salivary creatinine detection require sophisticated equipment, such as a Roche cobas® 6000 analyser, and operations in well-equipped laboratories with skilled personnel. Thus, the development of a simple, fast, and non-invasive POC assay for accurate detection of salivary creatinine detection for early-stage CKD diagnosis would be beneficial to patients suffering from CKD.

Noble metal nanoparticles, such as Ag NPs, have been widely used for colorimetric assays, due to their localised surface plasmon resonance (LSPR) effects in the visible light region.^11,15^ These nanoparticles produce a maximum optical extinction at their specific LSPR frequencies, which are highly sensitive to the particle size, shapes, degree of aggregation, and surroundings.^16^ Therefore, the colour change of the interparticle plasmonic coupling between Ag NPs could be applied for sensing purposes.^9^ In particular, they have shown the promising potential to be used in practical devices due to their high sensitivity, low production costs, and simple synthetic procedures, compared with other sensing platforms.^14^ For instance, He *et al.* described the citrate-capped gold nanoparticles (Au NPs) for the visual assay of urinary creatinine detection;^15^ Sittiwong *et al.* reported a method for detection of urinary creatinine by using the Au NPs coupled to an solid phase extraction process.^16^ Chio *et al.* developed a multi-spectroscopic detection method of urinary creatinine based on cucurbit^9^ uril and Au NPs.^17^ However, little work has been reported for the colorimetric detection of salivary creatinine using nanoparticles, as the salivary creatinine presents at a much lower concentration (0.003–0.4 mM), compared to the urinary creatinine (0.05–28.3 mM). In addition, pretreatment of saliva samples is typically required to reduce the matrix effects of saliva.

In this work, we report a facile colorimetric assay for the ultrasensitive detection of salivary creatinine. In this assay, citrate-capped Ag NPs act as the primary sensing probes which are initially light yellow in colour. The introduction of creatinine initiates ligand exchange between creatinine and the citrate ions on the surface of Ag NPs, resulting in the formation of creatinine-capped Ag NPs. The addition of Ag(i) ions then leads to Ag(i)–N and Ag(i)–π coordination interaction, causing the aggregation of the Ag NPs and a colour change of the colloid to bluish grey. The concentration of creatinine can be determined quantitatively by UV-Vis spectrometry or qualitatively by naked eyes. This Ag NPs-based colorimetric assay exhibits a low LOD down to 6.9 nM (*ca.* 0.78 ppb) in spiked artificial saliva with a dilution factor of 300, which is superior to many metal nanoparticle-based colorimetric assays for creatinine detection. The primary dependence in other works was placed on the hydrogen bond network formed between creatinine and silver nanoparticles. Conversely, our approach is credited to the coordination bond network formed between silver ions and creatinine on the surface of silver nanoparticles. This method holds the potential to be further developed into a POC diagnostic tool for CKD screening in resource-limited clinical settings.

## 2. Material and methods

### 2.1 Materials and apparatus

Silver nitrate (AgNO_3_) and sodium chloride (NaCl) were obtained from Fluka, Honeywell. Sodium borohydride (NaBH_4_), calcium nitrate (Ca(NO_3_)_2_), and sodium bicarbonate (NaHCO_3_) were from Acros. Sodium nitrate (NaNO_3_), potassium nitrate (KNO_3_), zinc nitrate (ZnNO_3_), iron(iii) nitrate (Fe(NO_3_)_3_), sodium phosphate (NaH_2_PO_4_), uric acid (C_5_H_4_N_4_O_3_), lactic acid, glucose (C_6_H_12_O_6_), urea (CO(NH_2_)_2_), creatinine (C_4_H_7_N_3_O), monobasic potassium phosphate (KH_2_PO_4_), and dibasic potassium phosphate (K_2_HPO_4_) were all purchased from Sigma-Aldrich. Magnesium nitrate (Mg(NO_3_)_2_), and ascorbic acid (C_6_H_8_O_6_) were purchased from Alfa Aesar. Trisodium citrate dihydrate (Na_3_C_6_H_5_O_7_·2H_2_O) was bought from LP Chemicals LTD. Artificial saliva was obtained from Glandosane. Deionised (DI) water was used to prepare all the solutions in this research. Unless otherwise stated, all chemicals are purchased at analytical grades.

UV-Vis spectrophotometer (PerkinElmer) was employed to study the localised surface plasmon resonance (LSPR) of Ag NPs and the creatinine detection. All samples were analysed in the spectral range of 350 nm to 600 nm, with a scanning resolution of 1 nm. 3.5 mL polystyrene cuvettes with 1 cm path length were used for characterisation. DI water served as a blank reference in this work.^18,19^ Malvern Zetasizer ZS90 Dynamic Light Scattering (DLS) has been used for particle size distribution measurement and zeta potential analysis. Transmission electron microscopy (TEM) characterisation of citrate-capped Ag NPs and creatinine treated Ag NPs was carried out on a JEOL JEM-2100Plus microscope at an accelerating voltage of 200 kV and an emission current of 120 μA. TEM samples were prepared by drop casting a diluted NP sample onto a holey carbon coated Cu grid (Agar Scientific).

### 2.2 Synthesis of citrate-capped silver nanoparticles

Citrate-capped Ag NPs was synthesised according to the modified Creighton method.^20^ AgNO_3_, NaBH_4_, and Na_3_C_6_H_5_O_7_·2H_2_O were employed as the metal precursor, reducing agent, and stabilising agent, respectively. Firstly, 30 mL of 2 mM cold NaBH_4_ aqueous solution was magnetically stirred in an ice bath. Under a continuous stirring at 450 rpm, 10 mL of 1 mM AgNO_3_ was added dropwise over 20 min. Then 5 mL of 1 mM sodium citrate aqueous solution was added into the mixture dropwise over 10 min. The citrate-capped Ag NPs colloid was stored under darkness overnight prior usage. According to the Beer–Lambert law,^21^ concentration of the as-synthesised citrate-capped Ag NPs was estimated based on an extinction coefficient of *ca.* 5.56 × 10^8^ M^−1^ cm^−1^ at 392 nm,^21^ and was calculated as *ca.* 2.3 nM.

### 2.3 Detection of creatinine

The detection of creatinine was carried out by the following processes. 150 μL of Ag NPs with the original concentration of *ca.* 2.3 nM was mixed with 200 μL creatinine solution at different concentrations from 0.01 μM to 10 μM for a 5 min room temperature incubation. 50 μL of 5 mM Ag(i) ions solution was added subsequently, after which a 30 min room temperature incubation was performed. Afterwards, 2 mL DI water was added into the above solution to dilute the mixture for UV-Vis measurement, as the extinction of the initial sample is too high in the UV-Vis region. The creatinine concentration in the mixture can be measured either by a visual observation when it is higher than *ca.* 4 μM, or a UV-Vis spectrophotometer when it is below 4 μM. In UV-Vis analysis, the extinction at 392 nm was used to estimate the extent of aggregation of Ag NPs, where a higher extinction indicates a more stable particle dispersion, while a lower extinction indicates a higher degree of aggregation. A calibration curve was then plotted to determine the relationship between the creatinine concentration and the extinctions at 392 nm. The standard deviation of blank samples was used to estimate the LOD, and sensitivity was estimated through both naked-eye colorimetry and UV-Vis spectrophotometry by using a reaction time of 30 min.

The following chemicals or ions were added to the system to study the selectivity of this assay, including Na^+^ from NaNO_3_, K^+^ from KNO_3_, Ca^2+^ from Ca(NO_3_)_2_, Mg^2+^ from Mg(NO_3_)_2_, Zn^2+^ from ZnNO_3_, Fe^2+^ from FeCl_2_, Fe^3+^ from Fe(NO_3_)_3_, NH_4_^+^ from NH_4_NO_3_, ascorbic acid, lactic acid, uric acid, glucose, urea, Cl^−^ from NaCl, PO_4_^3−^ from NaH_2_PO_4_, HCO_3_^−^ from NaHCO_3_, citrate from sodium citrate, and creatinine. All these solutions were prepared at the concentration of 100 μM. These solutions were mixed with 2.3 nM citrate-capped Ag NPs separately, and subsequently added with Ag(i) ions in final concentration of 625 μM, which will be kept constant throughout this work, unless otherwise stated. The mixtures were incubated at room temperature for 35 min before the UV-Vis measurement. Optical photos were taken to observe the colour changes with the naked eye, after the addition of the chemicals above.

As the chemical composition of human saliva varies from individuals at different times over the day, we employed an artificial saliva from Glandosane to estimate the feasibility of this assay for salivary creatinine testing. The chemical composition description is 1 wt% of carmellose sodium (C_8_H_16_NaO_8_), 3 wt% of sorbitol (C_6_H_14_O_6_), 0.12 wt% of potassium chloride (KCl), 0.08 wt% of NaCl, 0.005 wt% of magnesium chloride (MgCl_2_), 0.015 wt% of calcium chloride (CaCl_2_), and 0.034 wt% of K_2_HPO_4_ with a pH value of 5.8. The artificial saliva sample was diluted 300 times to reduce matrix effects and high viscosity.

## 3. Results and discussion

### 3.1 Working principle of creatinine detection using Ag NPs and Ag(i) ions

**Fig. 1:**
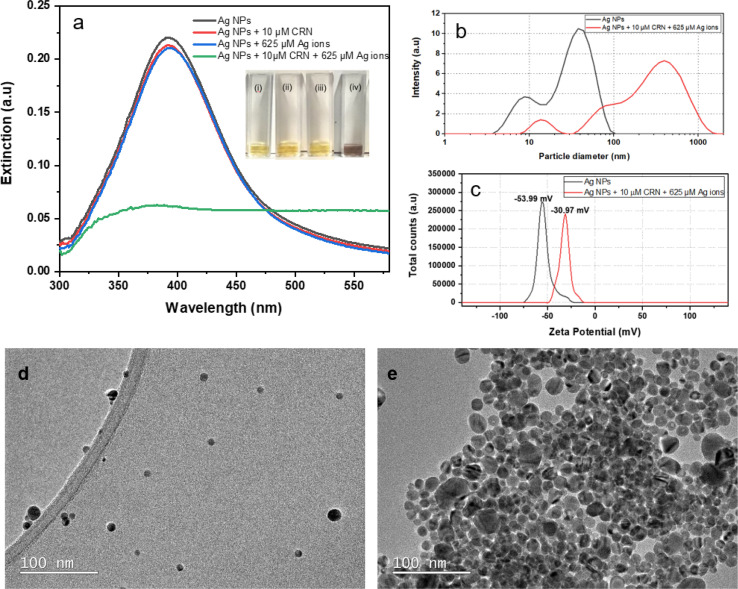
(a) UV-Vis spectra of citrate-capped Ag NPs in the presence of creatinine, Ag(i) ions, and both. Inset: optical images of the corresponding samples. (b) DLS analysis of particle size distribution and (c) zeta potential of citrate-capped Ag NPs in the absence and presence of creatinine and Ag(i) ions. TEM images of Ag NPs (d) in the absence and (e) in the presence of creatinine and Ag(i) ions. The concentrations for creatinine and Ag(i) ions are kept at 10 μM and 625 μM respectively for this set of characterisations.

To investigate whether the aggregation of citrate-capped Ag NPs is dependent on the synergistic influence of Ag(i) ions and creatinine, we prepared four groups of samples for comparison using UV-Vis spectroscopy (Fig. 1a). These groups includes: (i) DI water mixed with citrate-capped Ag NPs at a concentration of *ca.* 2.3 nM; (ii) 10 μM creatinine mixed with citrate-capped *ca.* 2.3 nM Ag NPs; (iii) Ag(i) ions solution mixed with citrate-capped Ag NPs in final concentration of 625 μM; (iv) 10 μM creatinine incubated with citrate-capped Ag NPs for 5 min and then 5 mM Ag(i) ions was added to give a final concentration of 625 μM. All mixtures were aged for 30 min and then diluted by adding 2 mL DI water with the fixed factor of 6 to ensure the extinction of the resulting dispersions was less than 1 for the optimal UV-Vis measurements. As shown in Fig. 1a, group (i) the citrate-capped Ag NPs shows the extinction peak at 392 nm and the light yellow colour in the corresponding optical image. Group (ii), the citrate-capped Ag NPs mixed with 10 μM creatinine shows the extinction peak shift to 401 nm; based on the optical image, no substantial colour change was evident. This suggests that the presence of a specific concentration of creatinine can induce the aggregation of Ag NPs by altering the interparticle forces through interactions with creatinine molecules.^16^ However, relying solely on this mechanism is insufficient for the development of a reliable colorimetric assay. Group (iii) displays the citrate-capped Ag NPs in the presence of Ag(i) ions. Similar to group (ii), the optical photo present a negligible colour change and minor extinction peak shift to 399 nm, as the introduction of Ag(i) ions caused by the surface charge screening. In group (iv), both creatinine and Ag(i) ions are present, the Ag NPs mixture exhibited a significant colour change to bluish grey. Additionally, there was a significant decrease in extinction at 392 nm. These observations provide strong evidence that the introduction of Ag(i) ions promotes the interaction between creatinine and Ag NPs.

DLS was used to analyse the size distribution of Ag NPs before and after the incubation with creatinine. Samples were prepared in the same way as for UV-Vis measurements. The hydrodynamic size measured by DLS refers to the effective combined size of a particle and its surrounding solvent molecules. Fig. 1b illustrates the hydrodynamic diameter distribution of the citrate-capped Ag NPs in the absence and presence of creatinine and Ag(i) ions. The resultant Ag NPs exhibit two major peaks, one at 9 nm and the other at 36 nm. The first peak corresponds to the rotational diffusion of the Ag NPs, indicating that they are slightly ellipsoidal.^22^ This produces no interference to the creatinine detection in this work. Upon the addition of creatinine and Ag(i) ions, the aggregated Ag NPs exhibit significant increase in hydrodynamic size. This direct evidence confirms the occurrence of Ag NPs aggregation from the introduction of creatine and Ag(i) ions. In addition, zeta potential of the citrate-capped Ag NPs before and after the addition of creatinine and Ag ions were shown in Fig. 1c. Generally, zeta potential of citrate-capped nanoparticles with values smaller than −30 mV are colloidally stable.^23^ The citrate-capped Ag NPs is this study have an average zeta potential value of −53.99 mV, indicating that these Ag NPs present a relative high degree of stability. Whilst the citrate-capped Ag NPs mixed with creatinine and Ag(i) ions present an average zeta potential value of −30.97 mV, indicating a notable decrease in colloidal stability of the Ag NPs.

TEM was used to characterise the Ag NPs before and after the creatinine and Ag(i) ions induced aggregation. The synthesised citrate-capped Ag NPs, with the monomodal extinction peak around 392 nm, have slightly elongated shapes as shown in Fig. 1d, consistent with the DLS data (Fig. 1b). Mainly individual particles are observed, which suggests that the Ag NPs are effectively stabilised by citrate-capped ligands against aggregation in the colloids. After mixing with creatinine and Ag(i) ions, significant aggregation of Ag NPs is observed as depicted in Fig. 1e, in line with the UV-Vis (Fig. 1a) and DLS (Fig. 1b).

**Fig. 2:**
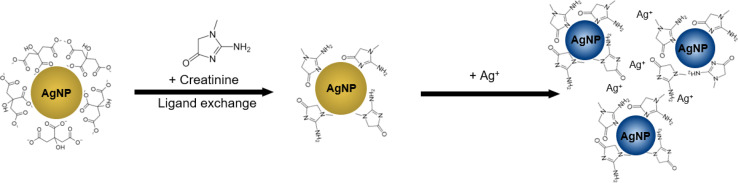
Working principle of the creatinine detection using citrate-capped Ag NPs with assistance of Ag(i) ions. Upon the addition of creatinine, the ligand exchange between creatinine and citrate occurs on the Ag NPs surface to form creatinine-capped Ag NPs. The subsequent introduction of Ag(i) ions leads to the formation of Ag(i)–N and Ag(i)–π coordination bonds with creatinine-capped on Ag NPs surface, resulting in the aggregation of Ag NPs and colour change from light yellow to bluish grey.

The colour of citrate-capped Ag NPs colloids originates from the localised surface plasmon resonance due to the coupling of incident photons and the collective oscillations of the conduction electrons.^24^ The resonance frequency can be modulated by tuning the nanoparticle size, shape, and arrangements,^25^ as well as the dielectric permittivity of the surrounding media.^26^ In our case, the colour change is triggered by aggregation of Ag NPs mediated by the dual binding affinity of creatinine for Ag NPs and Ag(i) ions. The working principle of our Ag NP-based colorimetric assay is summarised in Fig. 2. In particular, Ag NPs are initially capped by the negatively charged citrate ions, which prevent colloidal aggregation due to electrostatic repulsion. Since the electrostatic adsorption between Ag NPs surface and carboxylate group in citrate is weak, the latter can be replaced by ligands, such as melamine and luminol, that are able to form stronger interactions with Ag NPs surface^27^ due to the coordinated interaction between the nitrogen containing functional groups and nanoparticles.^15^ Similarly, creatinine has an aromatic ring structure and three nitrogen-containing binding sites.^32^ Upon the addition of creatinine to the citrate-capped Ag NPs, the creatinine will replace the citrate ions to form the creatinine-capped Ag NPs. The subsequent addition of Ag(i) ions will form Ag(i)–N and Ag(i)–π coordination bonds with creatinine,^28,29^ resulting in Ag NPs aggregation and colour change.

### 3.2 Optimisation of detection conditions

**Fig. 3:**
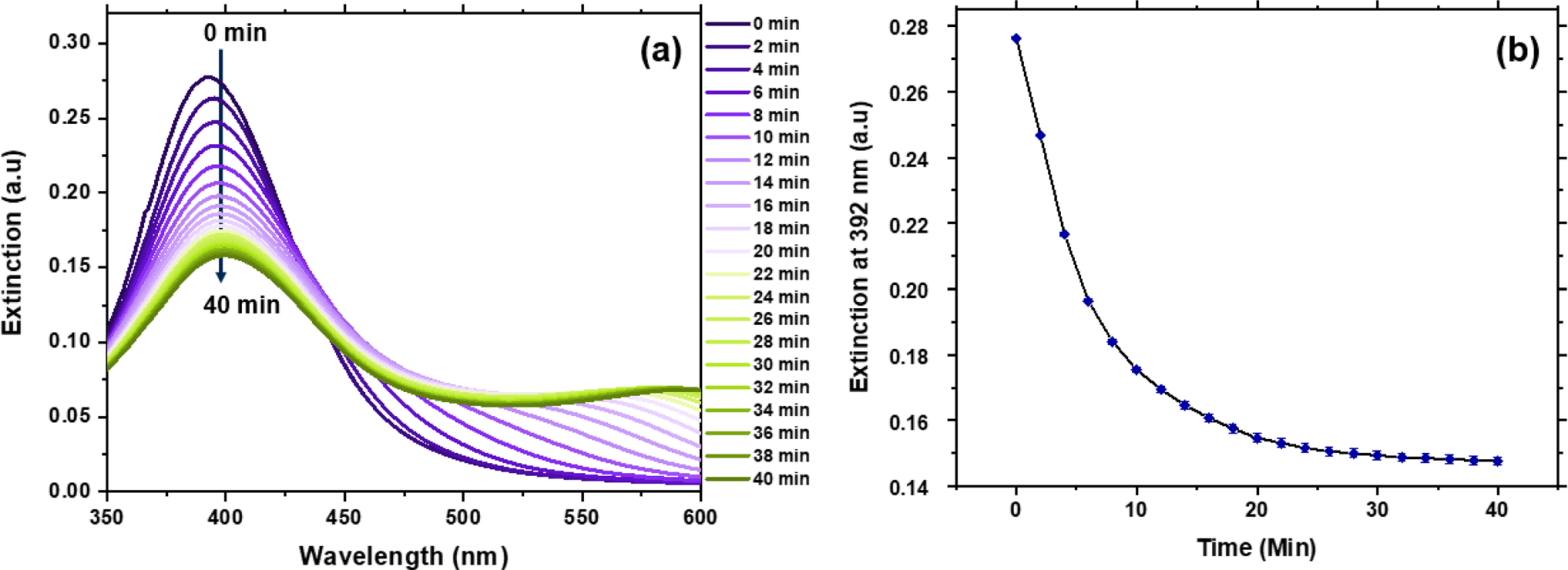
(a) Time-dependent UV-Vis extinction spectra of Ag NPs in the presence of 1 μM creatinine and 625 μM Ag(i) ions. (b) The corresponding evolution of extinction at 392 nm of the Ag NPs colloids after mixing with creatinine and Ag(i) ions.

The incubation time and pH condition for detection have been optimised in this study. To optimise the response time of this assay, we studied the effect of incubation time over the extinction (Fig. 3). With the increase of incubation time, the extinction at 392 nm decreased monotonically. The change of the extinction within 40 min were repeated three times, as shown in Fig. 3b. A standardised reference period of 30 min was selected for following detection, as there is no notable decrease observed after that period.

The pH value of the surrounding environment of Ag NPs is another potential factor affecting the sensitivity and accuracy of creatinine detection. It has been reported that the normal pH range of human saliva typically locates between 6.2 and 7.6. Furthermore, Tanoj *et al.* conducted a study on the end-stage renal disease patients and reported a salivary pH range of 5.39 to 7.59.^30^ Therefore, to assess the effect of pH on this assay, phosphate buffer solutions with pH values of 4.0, 5.0, 6.0, 7.0, and 8.0 were used to prepare media samples. 150 μL of Ag NPs was mixed with 200 μL of media sample at different pH values for 5 min. Subsequently, 50 μL of 5 mM Ag(i) ions solution was added to the mixture, followed by 30 min incubation. The resultant mixture was then diluted with 2 mL of DI water and used for UV-Vis measurement (see Fig. S1 in ESI†). The decrease of the extinction at 392 nm is under 0.035, while that of creatinine with concentration of 10 μM is more than 0.18 (Fig. 5), which means that the effect of pH on Ag NPs for creatinine detection is negligible between pH 4.0–8.0.

### 3.3 Colorimetric detection of creatinine in water

**Fig. 4:**
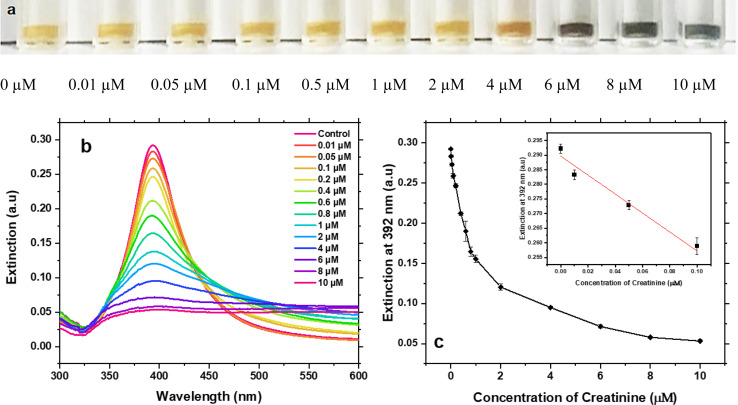
(a) Optical images of citrate-capped Ag NPs in the presence of 625 μM Ag(i) ions mixed with different concentration of creatinine. (b) Corresponding UV-Vis spectra of the samples. (c) Calibration curve of creatinine detection from 0.01 μM to 10 μM. Inset: linear fitting between the concentration range of 0.01–0.1 μM.

The performance of our assay for creatinine detection in DI water was tested under the optimised conditions. As creatinine concentrations increased, the colour of Ag NPs colloid gradually changed from light yellow to dark bluish grey (Fig. 4a) which become visible to naked eyes above 4 μM. Thus, this method can be used for naked-eye colorimetric detection when the creatinine concentration is higher than 4 μM.

Below this concentration, UV-Vis spectroscopy is required to characterise the LOD and the sensitivity of this assay. Fig. 4b shows the UV-Vis spectra of Ag NPs after adding different concentrations of creatinine. The extinction at 392 nm gradually decreased with increasing concentration of creatinine, whilst the extinction ranges from 500 nm to 600 nm gradually increased. The extinction at 392 nm was used to compare the aggregation of Ag NPs caused by different concentrations of creatinine. Fig. 4c presents the relationship between this extinction and the creatinine concentration. The results exhibit an exponential relationship when the concentration range is 0–10 μM with a pseudo-linear relationship between the concentration of 0.01 μM and 0.1 μM, which indicates the suitability of this method for low concentration creatinine detection. Next, a linear fitting was applied to the concentration range of 0.01–0.1 μM, as shown in inset of Fig. 4c. The fitting equation is shown in eqn (1) with a regression coefficient of 0.95.

1 *y* = (−0.32 ± 0.05)*x* + (0.29 ± 0.002)

where *y* is the extinction at 392 nm and *x* is the concentration of creatinine. The LOD equals to the average value of blank sample plus three times standard deviation of the blank.^31^ We thus determined the LOD of this assay to be 7.9 nM, which is much lower than the salivary creatinine concentration of 0.003–0.4 mM and the urine creatinine concentration of 1.768–37.04 mM.^7,32^ All the samples were repeated three times to estimate the reproducibility.

### 3.4 Assay selectivity

**Fig. 5:**
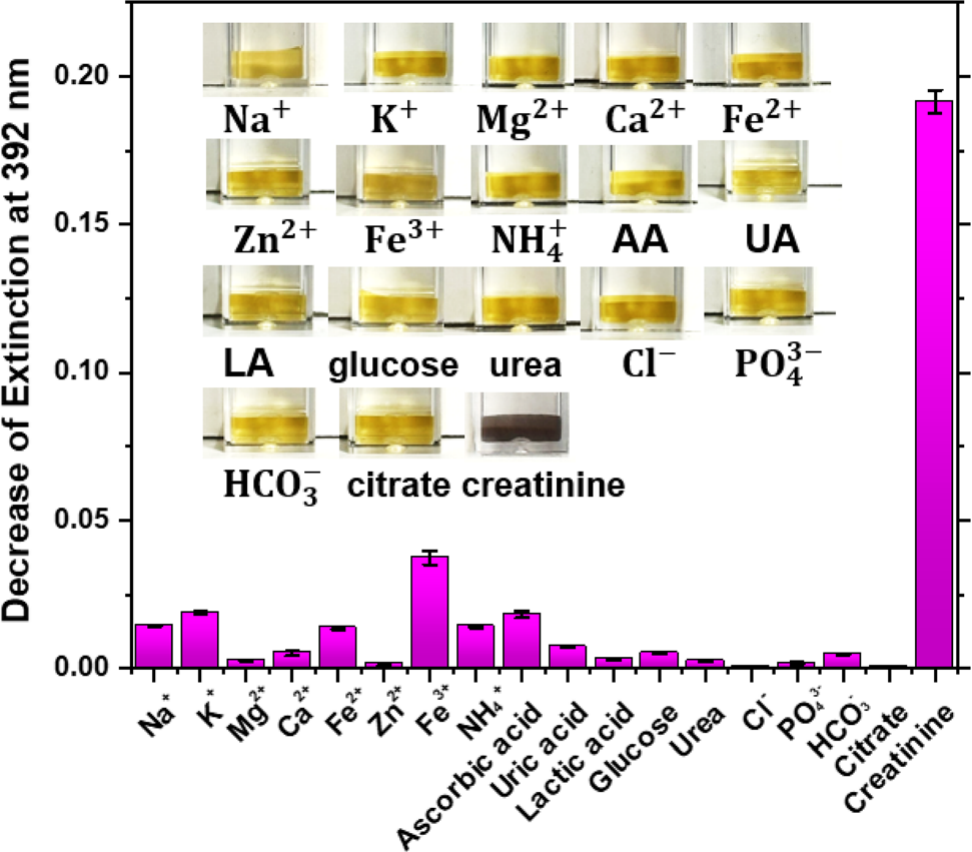
Plot of the decrease of extinction at 392 nm of citrate-capped Ag NPs in the presence of 625 μM Ag(i) ions plus 10 μM creatinine or 100 μM of different interference species in DI water, including Na^+^, K^+^, Mg^2+^, Ca^2+^, Fe^2+^, Zn^2+^, Fe^3+^, NH_4_^+^, ascorbic acid (AA), uric acid (UA), lactic acid (LA), glucose, urea, Cl^−^, PO_4_^−^, HCO_3_^−^, and citrate ions. Inset: optical images of the colour change caused by the corresponding interference molecules.

Diagnostic assay requires the high selectivity due to the complex composition in saliva samples, which can potentially interfere with creatinine detection. Thus, metal ions (Na^+^, K^+^, Mg^2+^, Ca^2+^, Fe^2+^, Zn^2+^ and Fe^3+^), non-metal ions (NH_4_^+^, Cl^−^, PO_4_^−^, HCO_3_^−^), trivial biomolecules (ascorbic acid, uric acid, lactic acid, glucose, urea), and target creatinine were used to investigate the selectivity of this assay. Apart from the creatinine, which is at 10 μM, all interfering chemicals are prepared at concentration of 100 μM. A blank sample was tested as a reference for data analysis. Only creatinine exhibited a significant colour change, as shown in the inset of Fig. 5, whilst other molecules caused negligible colour changes under the same experimental conditions. The decrease of the extinction at 392 nm was defined as the difference between the extinction of control and that of the samples.

It can be seen from Fig. 5 that only creatinine exhibits a decrease of extinction for up to 0.19, and the change of extinction of other chemicals are lower than 0.03. Fe^3+^ presents a decrease in extinction of 0.04, however, the Fe^3+^ concentration in human saliva after 300 dilution is 44.2 μM,^33^ which is about 2-fold lower than the concentration we used for this selectivity study. A systematic study of the effect of Fe^3+^ at different concentration to the detection of creatinine was provided in ESI, as Fig. S2.† In addition, the 300-dilution factor is necessary for reducing matrix effect when employing assay to saliva sample test. Theoretically, Cl^−^ will react with the extra free Ag(i) ions in the colloid and form AgCl sediment to interfere measurement. As mentioned above, the final concentration of additional Ag(i) ions is 625 μM, which is 13-fold higher than the Cl^−^ concentration of about 47 μM in resting parotid saliva after 300 dilution.^34^ Thus, the interference of Cl^−^ on the detection of salivary creatinine is negligible. In conclusion, this study demonstrated the good selectivity of this assay for the detection of salivary creatinine.

### 3.5 Detection of creatinine in saliva

**Fig. 6:**
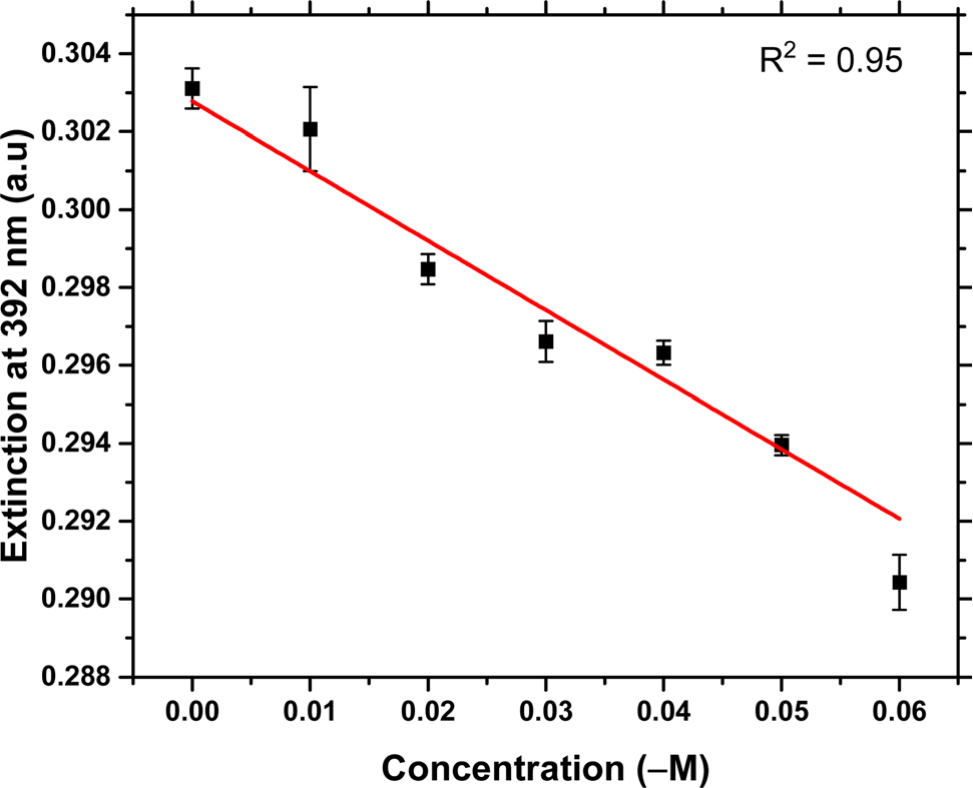
Calibration curve for the quantitation of creatinine in artificial saliva samples. The relationship between the extinction at 392 nm *versus* the creatinine concentration presents a negative linear correlation with regression coefficient of 0.95 between 0.01 and 0.06 μM.

Artificial saliva has been used to validate the creatinine detection of this assay. The high viscosity of undiluted artificial saliva could slow down the aggregation kinetics of colloid in the system and the matrix effect of artificial saliva may also affect the detection sensitivity. Therefore, it is important to dilute the artificial saliva before the detection. From literature,^35^ the concentration of human salivary creatinine is from 3 μM to 400 μM, and the cut-off point of creatinine concentration for the CKD diagnosis is 8.5 μM. Therefore, the dilution factor was selected to be 300, and the detection range of salivary creatinine is from 3 μM to 18 μM. The diluted artificial saliva samples were spiked with different concentrations of creatinine from 0.01 μM to 0.06 μM (0.028 μM is the diluted cut-off point). The calibration curve is shown in Fig. 6, with the linear fitting equation shown in eqn (2) and a regression coefficient of 0.95.

2 *y* = (−0.17863 ± 0.02)*x* + (0.30278 ± 0.0007)

where *y* is the extinction at 392 nm and *x* is the concentration of spiked creatinine. From the equation above, a negative linear relationship between the concentration and the extinction can be established, with a LOD calculated to be 6.9 nM. The error bars are calculated from the three independent repeating experiments. This result presents a strong potential of our method to the detection and quantitation of creatinine in human saliva.

## 4. Conclusion

In summary, we successfully developed a sensitive colorimetric assay for the detection of salivary creatinine with an extremely low LOD by adopting the ligand exchange between creatinine and citrate ions on the Ag NPs surface. The entire detection takes *ca.* 35 min and the citrate-capped Ag NPs changes from light yellow to dark bluish grey. UV-Vis spectroscopy is used to analyse the sample with low creatinine concentrations. This method has been experimentally proven to be effective for the detection of creatinine at both normal level and critical cut-off point for CKD diagnosis in artificial saliva samples. The method shows a great potential to be further developed as a POC analysis platform for the early-stage CKD diagnosis.

## Author contributions

J. H.: conceptualization, methodology, optimization, investigation, writing – original draft, writing – review & editing. M. S.: investigation, methodology, formal analysis, writing-review & editing. Gonzalez, A. R.: investigation, methodology. Y. K.: investigation, methodology. Y. W.: investigation, methodology, writing – review & editing. Y. L.: writing-review & editing. L. X.: writing-review & editing. M. W.: investigation, methodology. C. M.: investigation, methodology. A. D.: supervision, writing – review & editing. T. L.: supervision, methodology, writing – review & editing. B. L.: investigation, methodology, writing – original draft, writing – review & editing, supervision.

## Conflicts of interest

All authors declare that they have no known competing financial interests or personal relationships that could have appeared to influence the work reported in this paper.

## Abbreviations

AA: Ascorbic acid
Ag NPs: Silver nanoparticles
CKD: Chronic kidney disease
DLS: Dynamic light scattering
LA: Lactic acid
LOD: Limit-of-detection
LSPR: Localised surface plasmon resonance
POC: Point-of-care
TEM: Transmission electron microscopy
UA: Uric acid
UV-Vis: Ultraviolet-visible spectrophotometer

## Supplementary Material

RA-014-D3RA08736K-s001
