# Supplementary material for: Ultrasensitive colorimetric detection of creatinine via its dual binding affinity for silver nanoparticles and silver ions
Source: RSC Adv. 2024 Mar 18;14(13):9114–21. doi: 10.1039/d3ra08736k (PMC10946247; doi:10.1039/d3ra08736k)
Supplement: RA-014-D3RA08736K-s001 [file RA-014-D3RA08736K-s001.pdf]

Electronic Supplementary Material (ESI) for RSC Advances.
This journal is © The Royal Society of Chemistry 2024

Supporting Information

Ultrasensitive Colorimetric Detection of Creatinine via its Dual Binding Affinity for Silver Nanoparticles and Silver Ions

Jingle Huang [1], Maria Sokolikova [2], Antonio Ruiz-Gonzalez [1], Yingqi Kong [1], Yuxuan Wang [2], Yingjia Liu [3], Lizhou Xu [3, 4], Mingqing Wang [1], Cecilia Mattevi [2], Andrew Davenport [3], Tung-Chun Lee [1, *], Bing Li [1, *]

[1] *Institute for Materials Discovery, University College London, London, WC1H 0AJ, UK*

[2] *Department of Materials, Imperial College London, London, SW7 2AZ, UK*

[3] *Zhejiang University-Hangzhou Global Scientific and Technological Innovation Centre, Hangzhou 311200, China*

[4] *College of Biosystems Engineering and Food Science, Zhejiang University, Hangzhou 310058, China*

[5] *Department of Renal Medicine, Royal Free Hospital, University College London, NW3 2PF, UK*

*Corresponding authors

Email: bing.li@ucl.ac.uk; tungchun.lee@ucl.ac.uk

The effect of pH to the extinction at 392 nm was studied by adding 200 μL buffer solutions with pH of 4, 5, 6, 7 and 8 respectively to 150 μL of 2.3 nM Ag NPs dispersion. Then 50 μL of 5 mM Ag(I) ions solution was added to the mixture, followed by a 30-min incubation. The resulting mixture was then diluted with 2 mL of DI water and used for UV-Vis measurement. As shown in Figure S1, the maximum decrease of the extinction at 392 nm is under 0.035, while that of creatinine with concentration of 10 μM is more than 0.18 (as shown in Figure 5), meaning the effect of pH on Ag NPs for creatinine detection can be omitted.

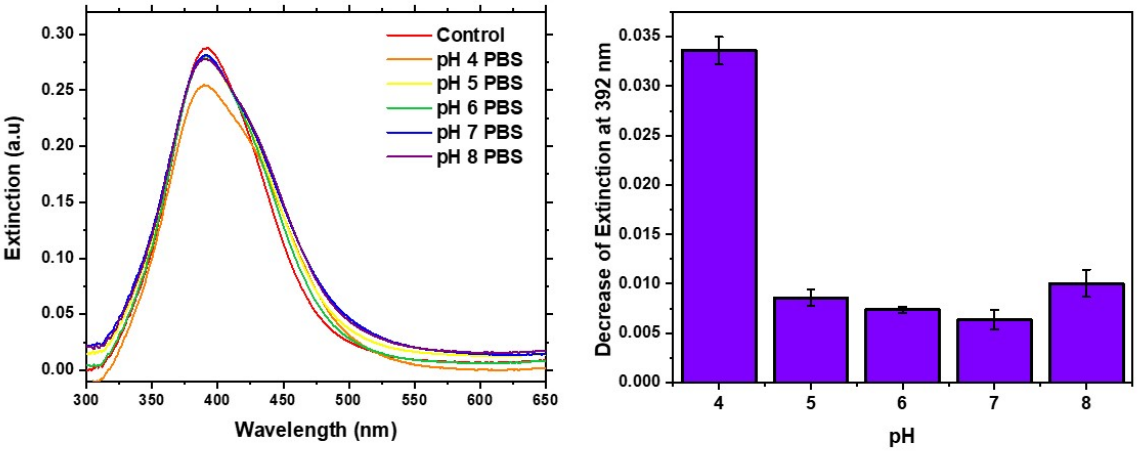

Figure S1. (a) The UV-Vis spectra of Ag NPs after adding buffer solutions with different pH values. (b) Corresponding decrease of the extinction at 392 nm. The decreased values are calculated as the difference between the extinction at 392 nm of blank sample and those of samples prepared with different pH values.

The physiological concentration of Fe (III) in human saliva, after a 300-fold dilution, ranges from 20 to 68.6 μM, with an average level of 44.2 μM. To understand their effects to the decrease of extinction at 392 nm, Fe(III) aquous solutions at the concentration of 20, 45, and 70 μM are prepared and the decreases of extinction at 392 nm are compared to creatinine (same concentration used in Fig.5 for seelectivity study). As shown in Figure S2 (a), the decrease of extinction of Fe (III) have been determined to be 0.00069, 0.0031 and 0.0069, respectively. This presents negligible effect to the detection of creatitine. Additionally, we performed the creatinine detection at their gradient concentrations (0, 0.02, 0.04, 0.06 μM) with the presence of Fe(III) at concentrations of 20 μM and 70 μM respectively (highest and lowest physiological concentration after dilution). As shown in Figure S2. (b) and S2. (c), we are able to achieve the linear detection with Fe(III) at 20 and 70 μM, suggesting it presence has insignificant impact on the detection of creatinine in artificial saliva.

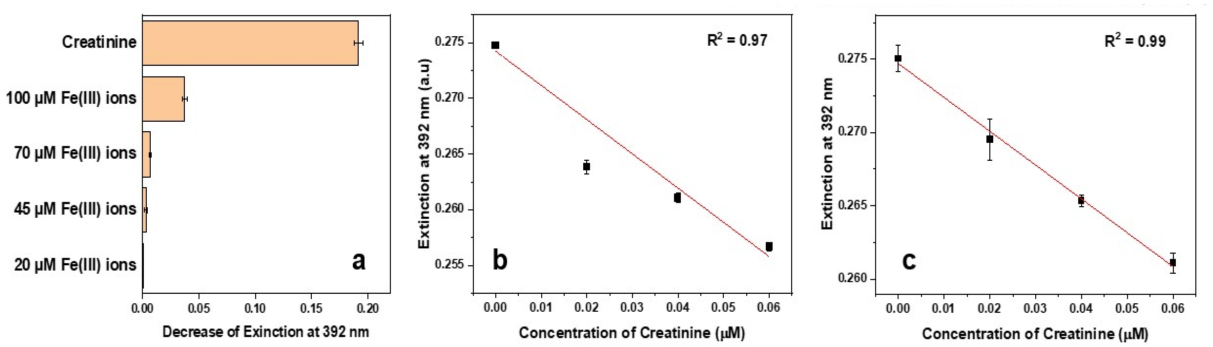

Figure S2. (a) Comparison of the decresase of extinction at 392 nm between different concetration of Fe(III) (20, 45, 70, and 100 μM) and creatinine at 10 μM. (b) Detection of creatinine in 300x diluted artificial saliva with presence of 20 μM Fe(III). Linear fitted as: y = (-0.30 ± 0.037) x + (0.27 ± 0.0012). (c) Detection of creatinine in 300x artificial saliva with presence of 70 μM Fe(III). Linear fitted as: y = (-0.23 ± 0.0093) x + (0.27 ± 0.00039).
